# Effect of family doctor contract services on non-communicable disease management among the elderly: a systematic review and meta-analysis

**DOI:** 10.3389/frhs.2025.1462806

**Published:** 2025-03-28

**Authors:** Rong Wu, Fei Fei, Tingting Lu, Jing Zhu, Dan Hu

**Affiliations:** ^1^ Department of Scientific and Technology, Nanjing First Hospital, Nanjing Medical University, Nanjing, China; ^2^ Department of Oncology, Nanjing First Hospital, Nanjing Medical University, Nanjing, China; ^3^ Division of Medical Affairs, The Affiliated Jiangning Hospital of Nanjing Medical University, Nanjing, China; ^4^ Division of Medical Affairs, Jiangning Clinical Medical College of Jiangsu Health Vocational College, Nanjing, China; ^5^ School of Health Policy & Management, Nanjing Medical University, Nanjing, China; ^6^ Creative Health Policy Research Group, Nanjing Medical University, Nanjing, China

**Keywords:** family doctor contract service, health management, non-communicable diseases, the elderly, meta-analysis

## Abstract

**Objective:**

The aim of this meta-analysis was to examine the effect of family doctor contract service on managing non-communicable diseases (NCDs) among elderly patients.

**Methods:**

Chinese and English articles published up to 15 July 2022 were systematically searched. Relevant randomized controlled studies (RCTs) were extracted from seven databases: PubMed, Coherence, Embase, Web of Science, CNKI, Wanfang Data, and WeiPu. All these studies have evaluated the effect of family doctor contract services on chronic disease management among the elderly. A meta-analysis was conducted using either random or fixed effects. Mean difference and risk ratio were used to analyze quantitative and qualitative data, respectively.

**Results:**

We identified that 25 independent studies, involving 4,046 elderly patients with chronic diseases across China, were eligible for meta-analysis. The results from these RCTs indicated that family doctors could disseminate knowledge about NCDs to elderly patients, improve their disease management abilities (including drug compliance, healthy diet, regular exercise, non-smoking, and non-drinking), lower blood pressure and blood glucose levels, reduce BMI, and increase quality of life and patient satisfaction (*P* < 0.05).

**Conclusion:**

Family doctor contract services could improve health management for elderly patients with NCDs and should be promoted in China.

## Background

Global population aging has picked up its pace. By 2050, the number of people aged 60 years or older is expected to exceed 2 billion (1). In 2021, 14.2% of China's population was aged 65 years and older, an increase from 7% two decades earlier. In May 2020, the UN General Assembly declared the period from 2021 to 2030 as the Decade of Healthy Aging, emphasizing the health concern regarding older adults. Non-communicable diseases (NCDs) are common among the elderly, and their prevalence keeps on increasing (2, 3). In 2016, NCDs were responsible for approximately 40.5 million out of 56.9 million global deaths (71%). Of them, an estimated 23.6 million (58%) were individuals aged 70 years and older (4). NCDs pose a significant threat to the health of the elderly (5, 6).

Health management is crucial for preventing and treating NCDs in elderly individuals. The demand for family medical services has increased in recent years (5, 7). Most patients with NCDs, particularly the elderly, lack knowledge about their conditions and often exhibit lower compliance and self-management abilities (8, 9). In response, various interventions have been developed to manage NCDs among the elderly, including “Internet + chronic disease” management and community home care (10). Among them, family doctor contract services have shown positive effects on NCD management (11–13). The WHO Implementation Road Map 2023–2030 emphasizes that various partners and stakeholders should collaborate to ensure universal access to NCD services and integrate these services into a primary healthcare framework. The 2030 Healthy China Plan also envisions the primary healthcare system as a means of addressing the burden of chronic NCDs (14, 15).

At present, more than 50 countries and regions have introduced a family medical service system, which has achieved remarkable outcomes in improving the health of the general population, reducing medical expenses, and allocating medical resources. This system offers comprehensive guidance and medical services to both patients and their family members for managing NCDs (13, 16). Family doctor contract services have been implemented in China for many years, but little is known about their potential impact on NCD management among elderly patients.

Here, we conducted a meta-analysis to evaluate the direct and indirect effect of family doctor contract services on NCD management across five aspects, including health education, self-management, physiological health, quality of life, and satisfaction. Our findings are expected to provide theoretical guidance for improving NCD management among the elderly in China.

## Methods

### Search strategy

We identified, selected, and analyzed randomized controlled trials (RCTs) that assessed the impact of family doctor contract services on the management of NCDs in the elderly, following the guidelines of the Preferred Reporting Items for Systematic Reviews and Meta-Analyses (PRISMA) (17). Using the systematic search strategy, we searched the following English and Chinese literature databases as of 15 July 2022: PubMed, Cochrane, Embase, Web of Science, CNKI, Wanfang Data, and WeiPu. We included only studies published in English and Chinese prior to 15 July 2022. The search terms included a combination of subject and free words. To maximize the coverage of our study, we also reviewed the references cited in the identified RCTs for additional relevant studies. The retrieval strategies are as follows (Table 1).

**Table 1:** Retrieval strategies.

Search	Search terms
#1	Family doctor OR primary care doctor OR general practitioner OR primary doctors OR family physician OR primary medical workers OR primary health worker OR family doctors OR village doctor OR family physicians
#2	Chronic disease OR comorbidity OR multimorbid OR complicated OR disease OR multiple OR TNF OR multi-morbid OR illness OR chronic OR chronic disease
#3	Ageing OR age related OR the elderly OR aged OR old people OR elder
#4	#1 AND (#2 OR #3)
#5	Effect OR effectiveness OR consequence

### Selection criteria

Inclusion and exclusion criteria were determined according to the Principles of the Cochrane Systematic Review Manual (PICOS).

The inclusion criteria were as follows:

(1) Population: The participants were elderly patients with chronic diseases.
(2) Interventions: The study provided a clear description of family doctor contract service.
(3) Comparison: After a period of intervention with family doctor contract services, relevant indicators were measured and compared between the intervention group and the control group.
(4) Outcomes: The indicators measured after intervention by a family doctor were related to one or more of the following aspects: health education, self-management, physiological health, quality of life, and safety.
(5) Design: The study followed a randomized controlled trial design.

The exclusion criteria were as follows:

(1) Population: The research participants were not elderly or did not have NCDs.
(2) Interventions: Studies did not use family doctor contract services as an intervention measure or did not provide a clear description of the intervention.
(3) Outcomes: After a period of intervention, none of the above-mentioned five aspects were measured.
(4) Design: The studies were reviews, meeting summaries, case reports, animal experiments, or other non-RCTs.

### Data extraction

Two reviewers independently extracted data from the full texts of potentially eligible articles, including the first author, publication year, study period, study location, sample size, types of chronic diseases, and evaluation indices, all following uniform criteria for each screening step. The reviewers resolved any inconsistencies through discussion. After reaching a consensus at each screening stage, the reviewers proceeded to the next step.

First, we imported all literature from the databases into Endnote X9 (Thomson Reuters, New York, NY, USA) to automatically detect and remove duplicates. Second, after two rounds of manual screening, we identified the RCTs that met our criteria. In the first round of screening, we reviewed the titles and abstracts of articles, excluding those unrelated to our study topic or research type; in the second round, we examined the full texts and checked them against the remaining criteria.

### Quality assessment of included studies

Using Review Manager 5.4 software, literature quality was evaluated based on the bias risk assessment tool outlined in the Cochrane Reviewers Handbook, involving random sequence generation, allocation concealment, blinding of participants and personnel, blinding of outcome assessment, incomplete outcome data, selective reporting, and other biases. All contents were evaluated from three aspects: “low risk,” “unclear risk,” and “high risk.”

### Statistical analysis

The effectiveness of family doctor contract services was evaluated across five aspects: health education, self-management, physiological indicators, quality of life, and safety. Heterogeneity was assessed using the Cochran *Q*-test, *P*-value, and *I*^2^ statistics. A *P*-value of less than 0.1 indicated statistically significant heterogeneity. We employed both fixed-effects and random-effects models to estimate the effectiveness of the five aspects. We used mean difference (MD) with confidence intervals (CI) to compare continuous variables and risk ratio (RR) with CI for categorical variables. When *I^2^* statistics exceeded 50%, the random-effects model was applied. All analyses were conducted using Review Manager 5.4.

## Results

### Study selection

**Figure 1:**
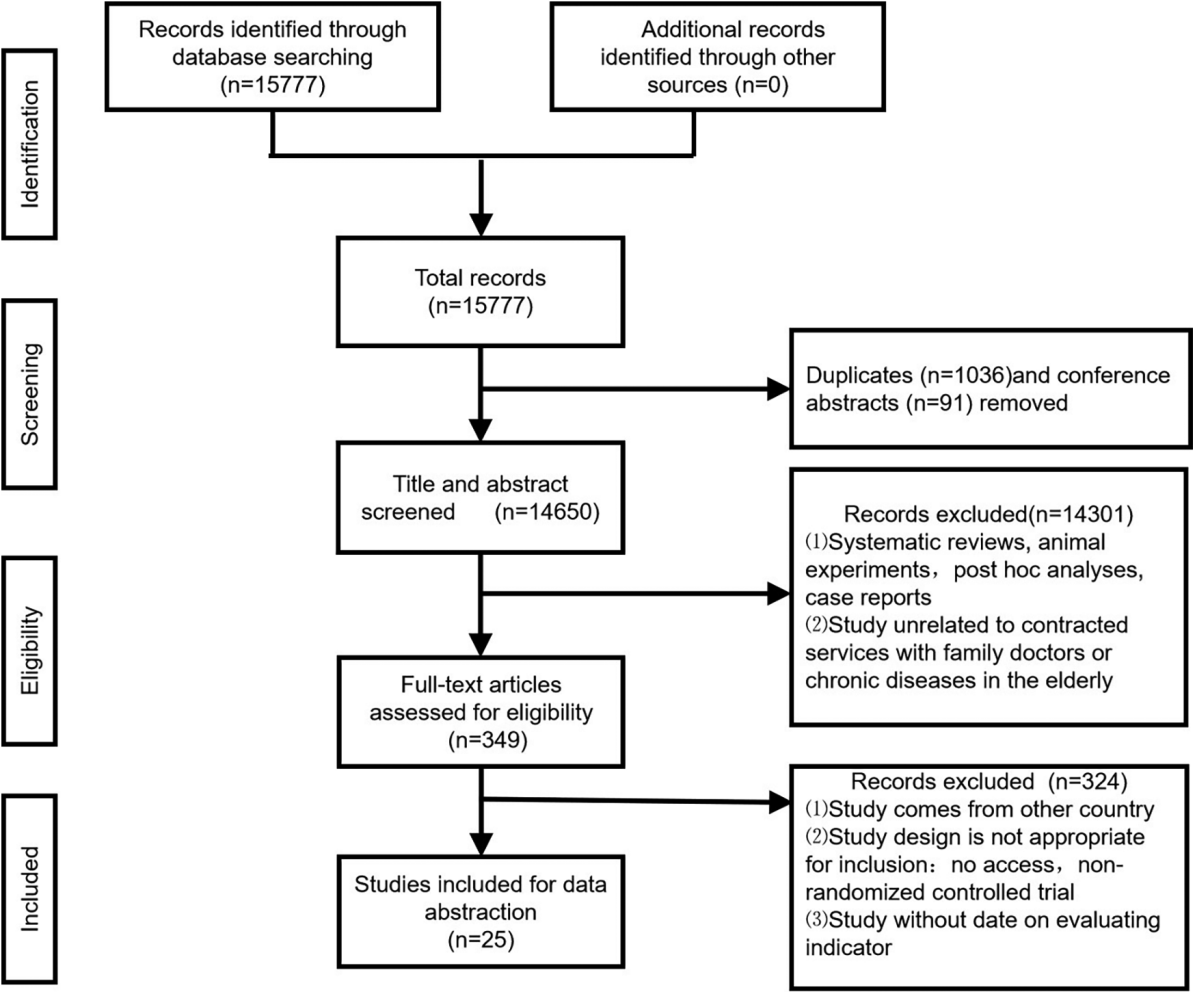
Flow of article selection for the systematic review.

We searched a total of 15,777 records across various databases: 1,911 from PubMed, 5,201 from Cochrane, 887 from Embase, 6,786 from Web of Science, 698 from CNKI, 54 from Wanfang, and 240 from WeiPu. After removing duplicates and irrelevant records, we screened 14,650 entries based on their titles and abstracts. Subsequently, we conducted a full-text review for 349 articles. Among these, 324 articles were eliminated due to outcomes from other countries, other designs, or missed data on variables. Ultimately, 25 studies were included in this study (Figure 1).

### Study characteristics

Table 2 outlines the key characteristics of the 25 independent studies included in this meta-analysis. These studies were conducted across 10 provinces in China from 2012 to 2021. The studies involved a total of 4,046 participants, with a median sample size of 108 and a range from 60 to 500. Twenty-one studies were conducted in eastern China, two in central China, and two in western China. Multiple variables might be used in one study. Five studies evaluated the effect on health education, 16 assessed the effect on self-management ability, 16 studies evaluated the effect on physiological health, 5 assessed the effect on quality of life, and 7 examined the effect on patient satisfaction.

**Table 2:** Characteristics of the 25 included studies.

Authors	Location	Year	Sample size of the intervention group (qualified rate %)	Sample size of the control group (qualified rate %)	Type of chronic disease	Aspect 1: health education	Aspect 2: self-management	Aspect 3: physiological health	Aspect 4: quality of life	Aspect 5: patient satisfaction
Liu (18)	Jiangsu	2020	36	36	Hypertension			√		
Wu (19)	Guangdong	2019	215	215	Hypertension			√		
Hu (20)	Guangdong	2021	54	54	Hypertension/diabetes/coronary heart disease			√		√
Lin et al. (21)	Shanghai	2019	53	53	Hypertension		√	√		
Huang et al. (22)	Guangdong	2021	59	58	Hypertension with hyperuricemia		√	√		
Li (23)	Guangdong	2019	79	79	Hypertension			√		
Liu et al. (24)	Guangdong	2020	65	65	Diabetes		√	√		
Mo et al. (25)	Guangxi	2020	42	41	Diabetes			√		
Feng et al. (26)	Guangdong	2021	60	60	Diabetes			√	√	√
Peng et al. (27)	Guangdong	2020	30	30	Hypertension			√	√	
Huang (28)	Shanghai	2020	42	41	Hypertension with diabetes		√	√	√	
Ye (29)	Guangdong	2021	43	43	Hypertension		√	√		
Li et al. (30)	Neimenggu	2018	41	41	Chronic obstructive pulmonary disease (COPD)		√			√
Luo (31)	Guangdong	2018	68	62	Hypertension/cardio cerebrovascular disease/diabetes					√
Li et al. (32)	Hunan	2017	53	53	Hypertension/hyperlipidemia/diabetes		√			√
Zhao et al. (33)	Shanghai	2020	246	246	Hypertension		√	√		
Guo et al. (34)	Shandong	2020	48	46	Hypertension/diabetes/coronary heart disease/osteoarthrosis		√		√	
Chen (35)	Guangdong	2018	100	100	All types	√	√			√
Hu et al. (36)	Henan	2012	43	43	All types		√			
Huang et al. (37)	Guangdong	2018	200	200	All types		√		√	
Lin et al. (38)	Guangdong	2017	250	250	All types	√	√			√
Pan et al. (39)	Guangdong	2020	65	63	All types	√		√		
Tan et al. (40)	Guangdong	2017	53	53	Hypertension		√	√		
Xia et al. (41)	Hainan	2021	59	56	All types	√	√			
Yuan (42)	Beijing	2021	32	32	All types	√	√	√		

### Study quality

**Figure 2:**
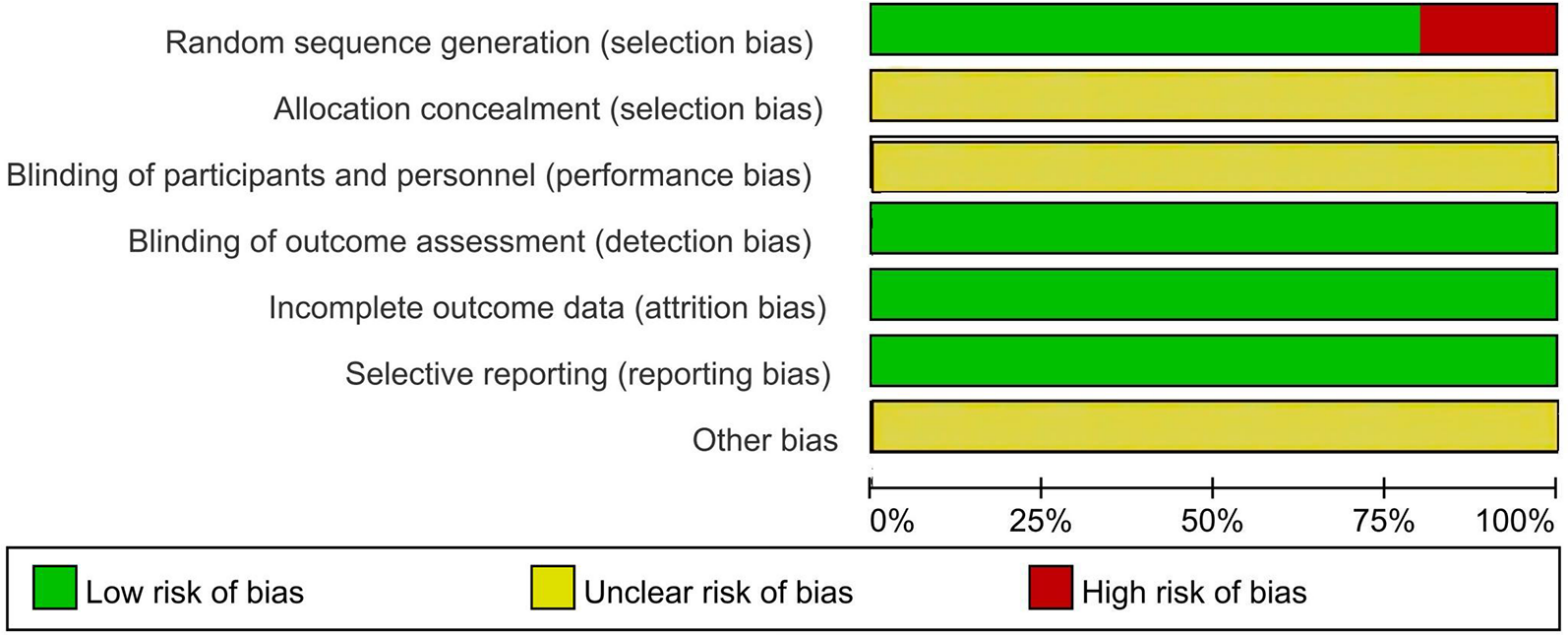
Risk of bias graph for all included studies.

**Figure 3:**
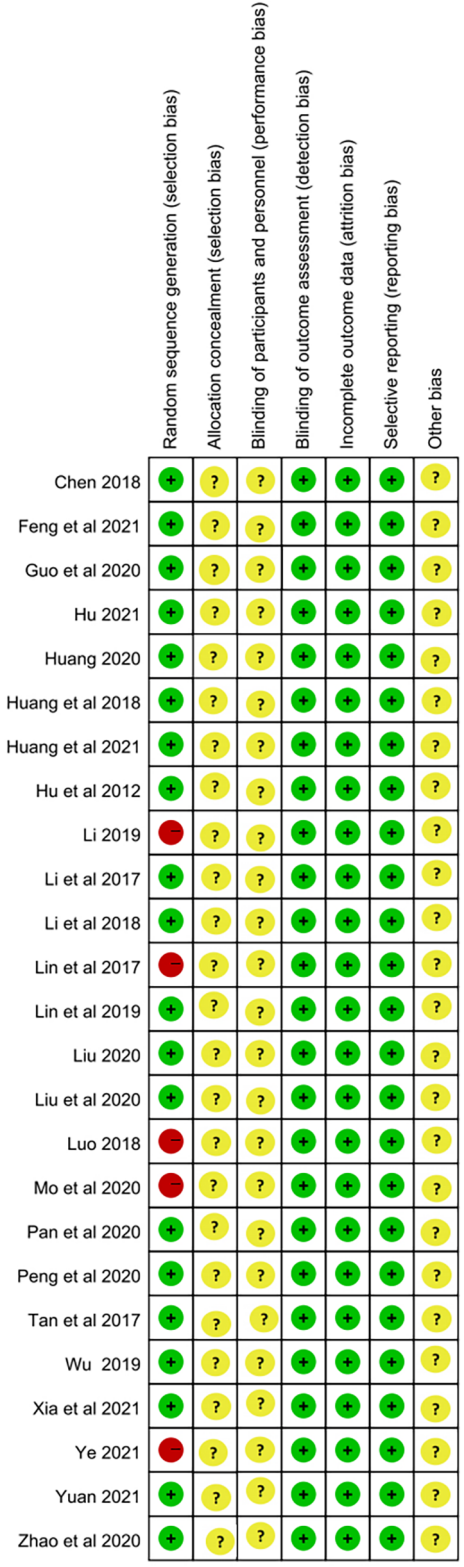
Risk of bias summary for all included studies.

Regarding random sequence generation, 20 studies explicitly used random allocation methods, while 5 studies considered both the availability of family doctor contract services and the willingness of the study population to participate, resulting in a “high-risk” rating. Since family doctor contract services involved on-site interventions, their implementation largely depended on participant cooperation and the feasibility of the intervention. Considering the comparable baseline data across various groups, we found the “high-risk” designation to be justified. During the intervention, participants must sign up for family health services, which makes it nearly impossible to conceal the allocation scheme or blind the participants. Therefore, none of the 25 studies discussed the “allocation concept” or “blinding of participants and personnel.” All studies employed blinding in data analysis, indicating that the risk of outcome assessment bias was low. Although some studies reported missing data, the amount was minimal and did not significantly impact the effect size. Consequently, all studies were considered at low risk for incomplete outcome data. All studies reported pre-specified research indicators and were considered to have a low risk of “selective reporting.” No studies mentioned any content related to “other biases.” Based on a comprehensive analysis, we believed that the quality of the included studies was relatively high, primarily due to their reliance on participant cooperation and transparent intervention methods (Figures 2, 3).

### Health education

**Figure 4:**
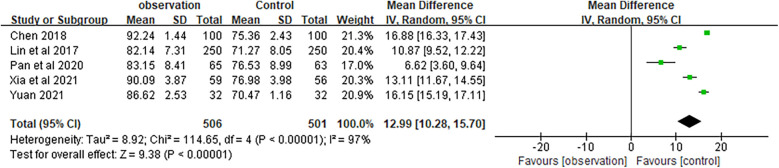
Forest plots of mastery of health knowledge.

The impact of health education was assessed using a health knowledge questionnaire. All five studies utilized a 100-point questionnaire. Higher scores reflected a better understanding of chronic NCDs among the elderly. These five studies reported a combined MD of 12.99 (95% CI 15.19–17.11) and a combined effect test statistic of *Z* = 9.38 (*P* < 0.01) (Figure 4). These results indicated that elderly patients who received family doctor contract services had a better knowledge of chronic NCDs than those who did not. This difference was statistically significant.

### Self-management

**Figure 5:**
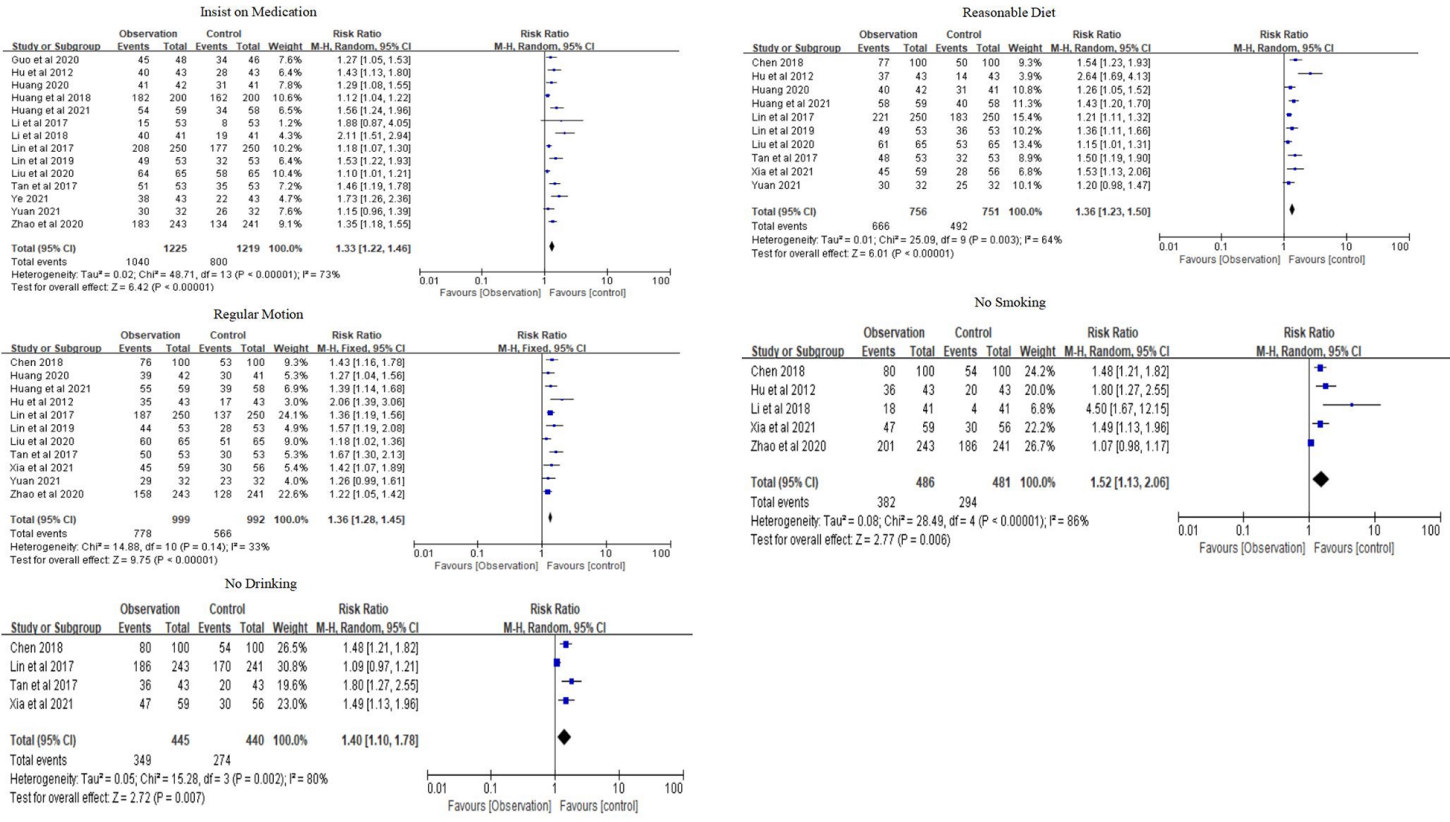
Forest plots of self-management.

Self-management ability was assessed using five indicators: medication compliance, healthy diet, regular exercise, no smoking, and no drinking. All studies were evaluated based on the rate of each item. The meta-analysis of medication compliance was based on 14 studies, with a combined RR of 1.33 (95% CI 1.22–1.46) and a combined effect *Z* of 6.42 (*P* < 0.01). The meta-analysis of a healthy diet was based on 10 studies, with a combined RR of 1.36 (95% CI 1.23–1.50) and a combined effect *Z* of 6.01 (*P* < 0.01). The meta-analysis of regular exercise was based on 11 studies, with a combined RR of 1.36 (95% CI 1.28–1.45) and a combined effect *Z* of 7.97 (*P* < 0.01). The meta-analysis of no smoking was based on five studies, with a combined RR of 1.52 (95% CI 1.13–2.06) and a combined effect *Z* of 2.77 (*P* < 0.01). The meta-analysis of no drinking was based on four studies, with a combined RR of 1.40 (95% CI 1.10–1.78) and a combined effect test *Z* of 2.72 (*P* < 0.01) (Figure 5).

Overall, the family doctor contract service significantly increased the rates of medication compliance, healthy eating, regular exercise, and smoking cessation among elderly patients with chronic NCDs compared to the control group.

### Physiological health

**Figure 6:**
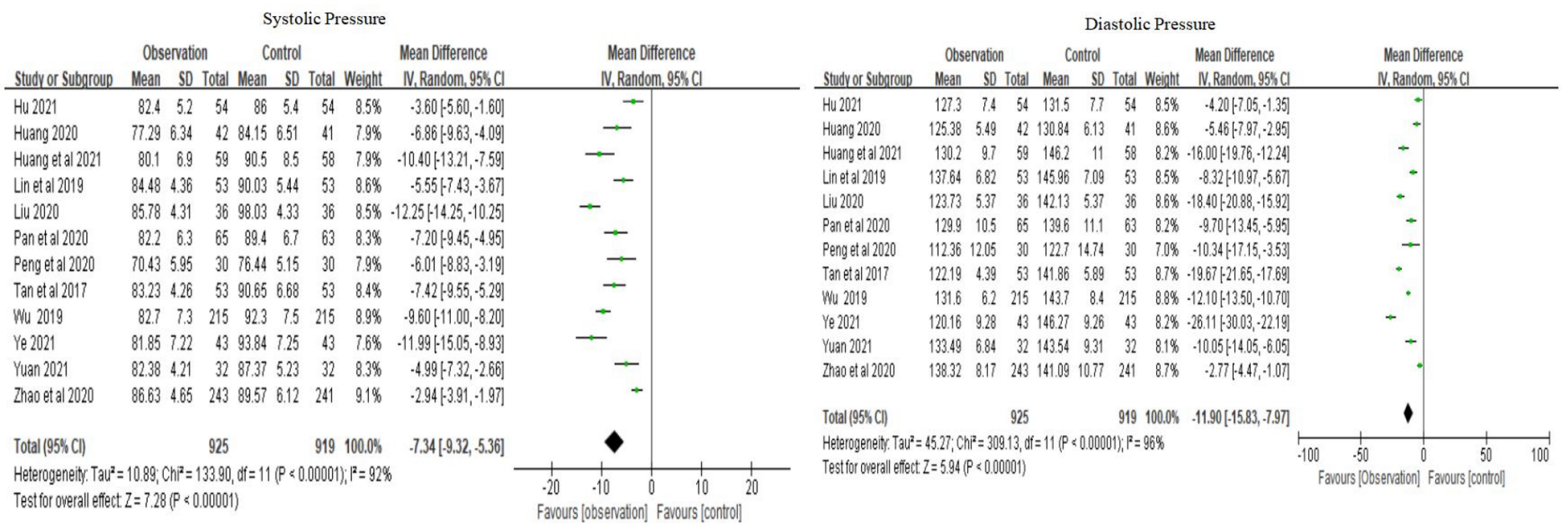
Forest plots of blood pressure.

Physiological health was evaluated using the following indicators: blood pressure, blood glucose, and BMI. The meta-analysis of diastolic pressure was based on 12 studies, with a combined MD of −7.34 (95% CI −9.23 to −5.36) and a combined effect *Z* of 7.28 (*P* < 0.01). The meta-analysis of systolic pressure was based on 12 studies, with a combined MD of −11.90 (95% CI −15.83 to −7.97) and a combined effect *Z* of 5.94 (*P* < 0.01) (Figure 6). Altogether, the family doctor contract service significantly lowered blood pressure levels in elderly patients with chronic NCDs compared to the control group.

**Figure 7:**
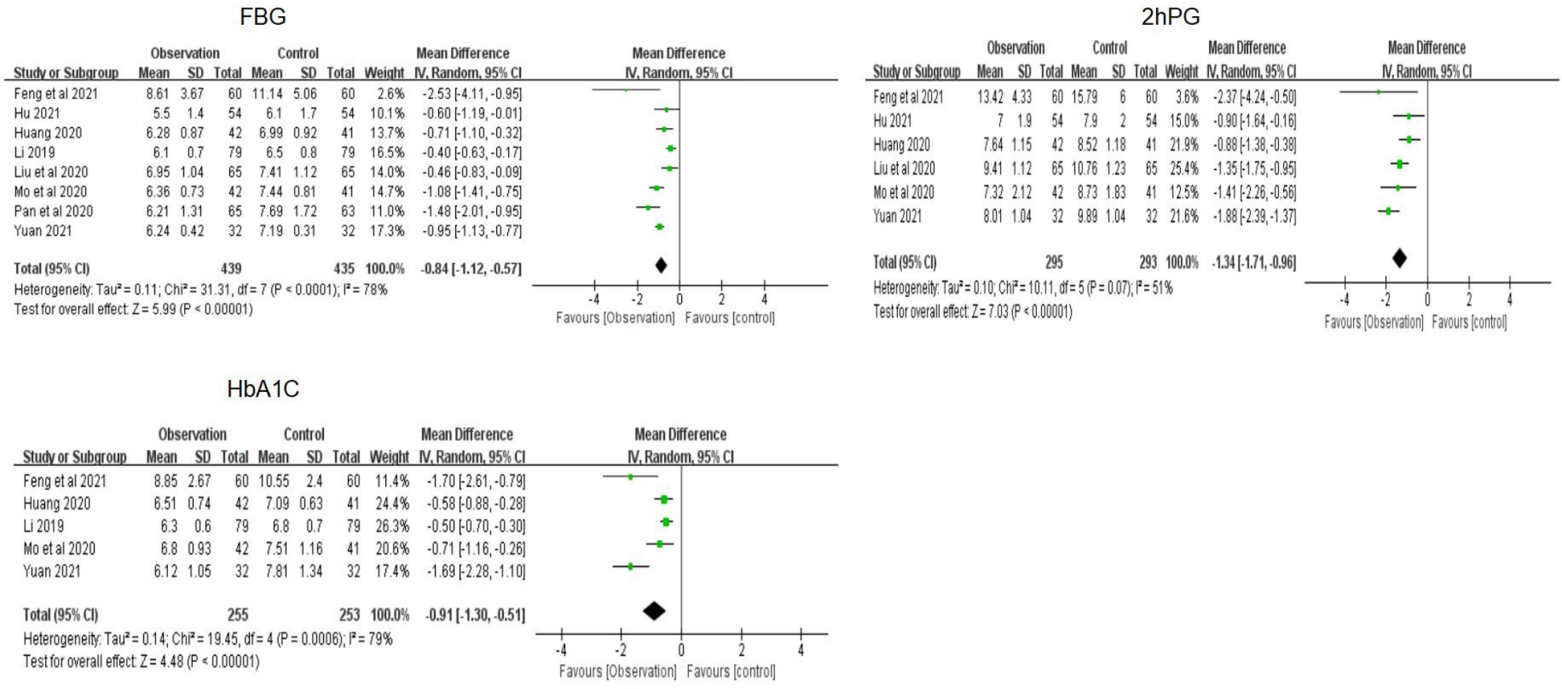
Forest plots of blood glucose.

The meta-analysis of fasting blood glucose (FBG) was based on eight studies, with a combined MD of −0.84 (95% CI [−1.12 to −0.57) and a combined effect *Z* of 5.99 (*P* < 0.01). The meta-analysis of 2-hour postprandial blood glucose (2hPG) was based on six studies, with a combined MD of −1.34 (95% CI −1.71 to −0.96) and a combined effect *Z* of 7.03 (*P* < 0.01). The meta-analysis of glycosylated hemoglobin (HbA1c) was based on five studies, with a combined MD of −0.91 (95% CI −1.30 to −0.51) and a combined effect *Z* of 4.48 (*P* < 0.01) (Figure 7). According to the three results, the family doctor contract service significantly decreased the blood glucose levels of elderly patients with chronic NCDs compared to the control group.

**Figure 8:**
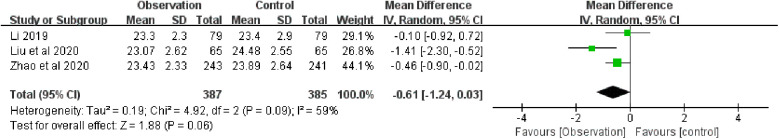
Forest plots of BMI.

The meta-analysis of BMI was based on three studies, with a combined MD of −0.61 (95% CI −1.24 to 0.03) and a combined effect test *Z* of 1.88 (*P* < 0.01), suggesting that the family doctor contract service significantly reduced BMI in elderly patients with chronic NCDs compared to the control group (Figure 8).

### Quality of life

**Figure 9:**
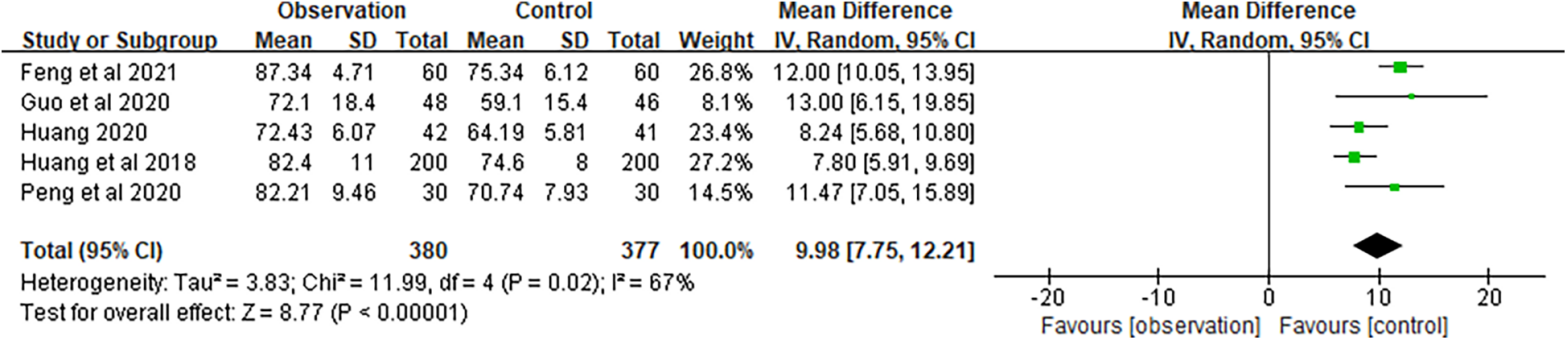
Forest plots of SF-36.

The quality of life was evaluated using the SF-36 scale, which has a full score of 100 points. A higher score indicates a better quality of life. The meta-analysis of SF-36 was based on five studies, with a combined MD of 9.98 (95% CI 7.75–12.21) and a combined effect *Z* of 8.77 (*P* < 0.01), suggesting that the family doctor contract service significantly enhanced the quality of life for elderly patients with chronic NCDs compared to the control group (Figure 9).

### Satisfaction

**Figure 10:**
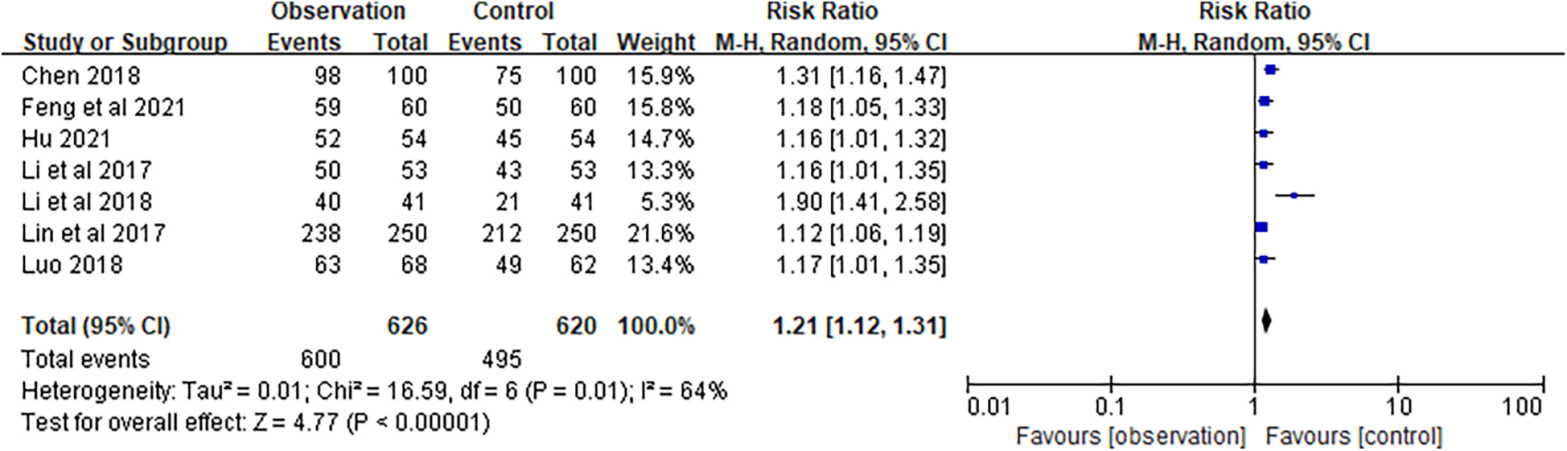
Forest plots of satisfaction.

Satisfaction was assessed based on the percentage of satisfied patients. The meta-analysis of satisfaction was based on seven studies, with a combined RR of 1.21 (95% CI 1.12–1.31) and a combined effect *Z* of 4.77 (*P* < 0.01), indicating that elderly patients with chronic NCDs reported a significantly higher degree of satisfaction with the family doctor contract service compared to the control group (Figure 10).

## Discussion

### Principal findings

This is a comprehensive systematic review and meta-analysis on the impact of family doctor contract services on elderly patients with NCDs in China. Of the 25 studies included in this study, 21 were conducted in eastern China. Relevant bibliometric data also indicate that most current studies concentrate on the populations in economically developed regions, such as Shanghai, Guangdong, and Beijing (43). We believe that the reporting bias is primarily due to the uneven distribution of family doctor contract services across regions in China. This disparity may arise from three main factors: first, eastern regions like Beijing and Shanghai, as pilot cities, have gained rich experience in implementing the family doctor contract service system; second, the eastern region has more abundant medical resources, and family doctors have stronger capabilities to offer services; and third, the economically developed eastern region invests much more into personnel, equipment, and information platforms necessary for implementing the family doctor system.

In the current comprehensive meta-analysis, we found that the effect of family doctor contract services on elderly patients with chronic NCDs was mainly evaluated using five aspects: health education, self-management, physiological health, quality of life, and satisfaction. In the 25 RCTs included, self-management and physiological health were most intensely studied. The reason may be that, currently, no effective measures are available for treating some chronic NCDs, which are mainly controlled through lifestyle interventions; meanwhile, physiological health can be evaluated using objective indicators. Second, we found that family doctor contract services had a positive effect on the management of chronic NCDs among the elderly. Both single and comprehensive RCTs showed that the family doctor contract service could enrich the NCD knowledge of elderly patients, enhance self-management ability (including medication compliance, healthy diet, regular exercise, no smoking, and no drinking), reduce blood pressure, blood glucose, and BMI, improve quality of life, and enhance satisfaction among elderly Chinese patients. In other countries, numerous studies have examined the effectiveness of family doctors in managing chronic diseases among the elderly. Rankin et al. (44) found that family doctor contract services can enhance the management of chronic diseases, such as lower back pain, in the elderly. Bennett et al. (45) reported that family doctors play multiple roles in addressing age-related hearing loss. In addition, Visvanathan et al. (46) discovered that having a family doctor can reduce the mortality rate of elderly individuals by 14%. These findings, together with our current research, illustrate the beneficial role of family doctors in managing chronic diseases among older adults.

### Strengths and limitations

Family doctor services emerged in the 20th century in foreign countries but were introduced as late as 2007 in Shanghai, China (47). In China, patients are likely to visit comprehensive and large hospitals for chronic NCDs. Studies have shown a decline in the number of elderly patients with chronic NCDs visiting grassroots hospitals in China (48). Compared with hospitals, family doctor contract services can optimize the allocation of medical resources and reduce medical expenses for Chinese residents, but their spread has seen a slow pace. The present study can provide evidence for promoting this service, as indicated by its evident effects in the five aspects.

However, this study has certain limitations. The first limitation is that the heterogeneity in the meta-analysis increased when the estimates were aggregated. This heterogeneity may be related to the differences in study methods, sample size, or regions. This heterogeneity may be overestimated when too few or too many eligible studies are included (49). Second, some effectiveness indicators were not evaluated in some studies; however, to perform a comprehensive evaluation, these studies were not excluded, which is a source of high heterogeneity. Third, to evaluate the effect using the five aspects, such as the mastery of health knowledge, the questionnaires might be differently designed by research institutes, which is also a source of high heterogeneity.

### Prospective

Future studies should focus on the family doctor contract service in central and western China. Second, evaluation tools for the effect of family doctor contract services should be unified, thus reducing the heterogeneity between studies. Finally, studies should also focus on the obstacles limiting the spread of family doctor contract services. One primary reason for the bias in this research report is the differing ability to implement family doctor services, which stems from the economic gap between eastern and central China. This highlights the importance of the family doctor service system in allocating limited medical resources effectively. Therefore, to promote the implementation of family doctor services, future research should include a cost–benefit analysis.

## Conclusion

Family doctor contract services are conducive to spreading knowledge about chronic NCDs in elderly patients, enhancing their self-management ability, and improving their physiological health, quality of life, and satisfaction. This service should be widely replicated in China.

## Data Availability

The original contributions presented in the study are included in the article and chart materials further inquiries can be directed to the corresponding authors.

## References

[1] Beard JR Officer A de Carvalho IA Sadana R Pot AM Michel JP The world report on ageing and health: a policy framework for healthy ageing. Lancet. (2016) 387(10033):2145–54. 10.1016/S0140-6736(15)00516-4 26520231 PMC4848186

[2] Chen M Xu L Si L Wang Z Jan S. Examining the level and distribution of catastrophic health expenditure from 2013 to 2018: a province-level study in China. Econ Model. (2023) 121:106233. 10.1016/j.econmod.2023.106233

[3] Ding D Lawson KD Kolbe-Alexander TL Finkelstein EA Katzmarzyk PT van Mechelen W The economic burden of physical inactivity: a global analysis of major non-communicable diseases. Lancet. (2016) 388(10051):1311–24. 10.1016/S0140-6736(16)30383-X 27475266

[4] NCDC. NCD countdown 2030: worldwide trends in non-communicable disease mortality and progress towards sustainable development goal target 3.4. Lancet. (2018) 392(10152):1072–88. 10.1016/S0140-6736(18)31992-5 30264707

[5] Figueiredo AEB Ceccon RF Figueiredo JHC. Chronic non-communicable diseases and their implications in the life of dependent elderly people. Cien Saude Colet. (2021) 26(1):77–88. 10.1590/1413-81232020261.33882020 33533865

[6] Wong WL Su X Li X Cheung CM Klein R Cheng CY Global prevalence of age-related macular degeneration and disease burden projection for 2020 and 2040: a systematic review and meta-analysis. Lancet Glob Health. (2014) 2(2):e106–16. 10.1016/S2214-109X(13)70145-1 25104651

[7] Feng Z Glinskaya E Chen H Gong S Qiu Y Xu J Long-term care system for older adults in China: policy landscape, challenges, and future prospects. Lancet. (2020) 396(10259):1362–72. 10.1016/S0140-6736(20)32136-X 34338215

[8] NCDC. NCD countdown 2030: pathways to achieving sustainable development goal target 3.4. Lancet. (2020) 396(10255):918–34. 10.1016/S0140-6736(20)31761-X 32891217 PMC7470795

[9] Fisher K Markle-Reid M Ploeg J Bartholomew A Griffith LE Gafni A Self-management program versus usual care for community-dwelling older adults with multimorbidity: a pragmatic randomized controlled trial in Ontario, Canada. J Comorb. (2020) 10:2235042X20963390. 10.1177/2235042X20963390 33117723 PMC7573753

[10] Kastner M Cardoso R Lai Y Treister V Hamid JS Hayden L Effectiveness of interventions for managing multiple high-burden chronic diseases in older adults: a systematic review and meta-analysis. CMAJ. (2018) 190(34):E1004–12. 10.1503/cmaj.171391 30150242 PMC6110649

[11] Jimenez Carrillo M Leon Garcia M Vidal N Bermudez K De Vos P. Comprehensive primary health care and non-communicable diseases management: a case study of El Salvador. Int J Equity Health. (2020) 19(1):50. 10.1186/s12939-020-1140-x 32252764 PMC7132977

[12] Kabir A Karim MN Billah B. The capacity of primary healthcare facilities in Bangladesh to prevent and control non-communicable diseases. BMC Prim Care. (2023) 24(1):60. 10.1186/s12875-023-02016-6 36864391 PMC9979470

[13] Yan C Yuan Y Zhao D Li J Fu P Chen Y Family doctor contract services and awareness of blood pressure measurement among hypertension patients: a cross-sectional study in rural Shandong, China. Front Public Health. (2022) 10:757481. 10.3389/fpubh.2022.757481 35372224 PMC8966041

[14] Fang EF Scheibye-Knudsen M Jahn HJ Li J Ling L Guo H A research agenda for aging in China in the 21st century. Ageing Res Rev. (2015) 24(Pt B):197–205. 10.1016/j.arr.2015.08.003 26304837 PMC5179143

[15] Nie Z Chen C Chen G Wang C Gan Y Feng Y Development and validation of a model to predict the contract service of family doctor: a national survey in China. Front Public Health. (2022) 10:750722. 10.3389/fpubh.2022.750722 35548082 PMC9082311

[16] Zomahoun HTV Samson I Sawadogo J Massougbodji J Gogovor A Diendere E Effects of the scope of practice on family physicians: a systematic review. BMC Fam Pract. (2021) 22(1):12. 10.1186/s12875-020-01328-1 33419398 PMC7796628

[17] Zhu Z Xie H Liu S Yang R Yu J Yan Y Effects of physical exercise on blood pressure during pregnancy. BMC Public Health. (2022) 22(1):1–13. 10.1186/s12889-022-14074-z 36096756 PMC9469521

[18] Miao L. Analysis of the effect of family doctor contract service health management on hypertension patients. Contemp Med. (2020) 18(19):174–6. 10.3969/j.issn.2095-7629.2020.19.124

[19] Minli W Xingzhen L Jiufeng S Wanyi D Wanfei Y. Analysis of the application effect of the improved health management model based on the contracted services of family doctors in the health management of hypertension patients. Intern Med. (2019) 14(2):215–7. 10.16121/j.cnki.cn45-1347/r.2019.02.26

[20] Xiaofang H. Evaluation on the application of family contract service model in community nursing for elderly chronic diseases. Chin Community Physicians. (2021) 37(6):143–4. 10.3969/j.issn.1007-614x.2021.06.071

[21] Linjun J Chunli Y. Study on the effectiveness of family doctor’s chronic disease outpatient service in the management of senile hypertension. Guizhou Pharm. (2019) 43(5):787–8. 10.3969/j.issn.1000-744X.2019.05.045

[22] Huihui H Wu C Jiejin F. Effect of family doctor service on blood pressure and blood uric acid level in patients with primary hypertension and hyperuricemia in community. Grassroots Med Forum. (2021) 25(8):1047–9. 10.19435/j.1672-1721.2021.08.005

[23] Rong L. Research on the Impact of Family Doctor Team Service on Self-management Ability and Health Status of Elderly Diabetes Patients in the Community Under the Medical Union Model. Guangzhou: Southern Medical University (2019).

[24] Lixia L Qingbiao L Bin S Baixian X. Effect of family doctor model in follow-up management of patients with type 2 diabetes. China Med Rec. (2020) 21(2):96–8. 10.3969/j.issn.1672-2566.2020.02.033

[25] Yan M Tiesheng Q Huisen H Rongdan Q Shuguang L Honghua F. The impact of family doctor contract service on self-management of empty nest elderly diabetes patients. Guangxi Med J. (2020) 42(16):2171–3. 10.11675/j.issn.0253-4304.2020.16.29

[26] Xiuling F Changyu L Huahong D. The application of the new model of family doctor contract service in diabetes patients. Gen Nurs. (2021) 19(7):1004–6. 10.12104/j.issn.1674-4748.2021.07.042

[27] Liyun P Na J Rongjiang Z. Evaluation of the application effect of family doctor contract service combined with family support in elderly chronic disease health management. Grassroots Med Forum. (2020) 24(29):4251–3. 10.19435/j.1672-1721.2020.29.068

[28] Xinlu H. The impact of family doctor contract management on elderly hypertension patients with type 2 diabetes living alone. Shanghai Pharm. (2020) 41(8):42–6. 10.3969/j.issn.1006-1533.2020.08.014

[29] Caihong Y. The effect of family doctor contract management on blood pressure control and self-management ability of elderly patients with essential hypertension in the community. Electron J Cardiovasc Dis Integr Tradit Chin West Med. (2020) 8(27):195–6. 10.16282/j.cnki.cn11-9336/r.2020.27.139

[30] Bing L Xinhou W Yufei C. The effect of family doctor responsibility system on the quality of life of patients with chronic obstructive pulmonary disease. China Emerg Med. (2018) 38(10):206–7. 10.3969/j.issn.1002-1949.2018.z1.195

[31] Jiehui L. The impact of family-contracted services on the quality of life of elderly patients with chronic diseases in the community. Nurs Pract Res. (2018) 15(17):146–7. 10.3969/j.issn.1672-9676.2018.17.059

[32] Aiqiong L Siyuan T Fen L Liping Q Wufei L Jirong L. Effect of family-contracted services on medication compliance of elderly patients with chronic diseases in the community. Nurs Res. (2017) 31(16):2005–7. 10.3969/j.issn.1009-6493.2017.16.023

[33] Xifang Z Jing W Hengna L Xue H. Evaluation on the effect of “1+1+1” contract management of hypertension patients by family doctors. J Cardiovasc Cerebrovasc Dis Integr Tradit Chin West Med. (2020) 18(10):1648–51. 10.12102/j.issn.1672-1349.2020.10.038

[34] Xi G Huiying J Liming Z XiuFen G Ruhong Q Haijing D. Health empowerment intervention for elderly patients with chronic comorbidities based on community family doctor system. J Nurs. (2020) 35(16):97–9. 10.3870/j.issn.1001-4152.2020.16.097

[35] Jiewen C. Analysis of the effect of family doctor’s contracted service mode on the prevention and treatment of chronic diseases of elderly patients in the community. Heilongjiang Tradit Chin Med. (2018) 47(5):91–2.

[36] Huihua H Yutian W Zhen Q Qian Z Haiying G Shumei G Study on the management effect of the “film doctor responsibility system” on elderly patients with chronic diseases in Zhengzhou China. Gen Med. (2012) 15(16):1861–3. 10.3969/j.issn.1007-9572.2012.06.026

[37] Guihao H Weili L. Discussion on the influence of family doctor’s contracted service on medication compliance of elderly patients with chronic diseases in the community. Mod Diag Treat. (2018) 29(3):493–4. 10.3969/j.issn.1001-8174.2018.03.078

[38] Peisen L Yanming Y Dongri C. Evaluation of the application effect of family doctor service in the health management of elderly chronic diseases. J Chronic Dis. (2017) 18(1):2–4. 10.16440/j.cnki.1674-8166.2017.01.002

[39] Jiaojiao P Xiaolong P Yun H Hongbo G. Discussion on the effect of family doctor team contract service on health management of elderly patients with chronic diseases. Abstr Worlds Latest Med Inf. (2020) 20(28):83–5. 10.3969/j.issn.1671-3141.2020.28.049

[40] Long T Xiaoxiong D Ligui Q. Effect of family doctor service management on elderly hypertensive patients in community. Clin Med Eng. (2017) 24(4):491–2. 10.3969/j.issn.1674-4659.2017.04.0491

[41] Yan X Beiying H Xiaoyan X Yunbo Z. The application value of family doctor contract service in the management of elderly patients with chronic diseases in the community. Lab Med Clin. (2021) 18(15):2262–4. 10.3969/j.issn.1672-9455.2021.15.036

[42] Zhanmin Y. Application effect of family doctor contract service in elderly chronic disease health management. Chin Mod Dr. (2021) 59(22):90–3.

[43] Mengyu C Qianqian Y Wenqiang Y Dongmei H Kui S Zhongming C Literature analysis on the implementation effect of family doctor contract service policy in China. Chin J Hosp Manag. (2019) 35(8):652–6. 10.3760/cma.j.issn.1000-6672.2019.08.009

[44] Rankin NM York S Stone E Barnes D Mcgregor D Lai M Pathways to lung cancer diagnosis: a qualitative study of patients and general practitioners about diagnostic and pretreatment intervals. Ann Am Thorac Soc. (2017) 14(5):742–53. 10.1513/AnnalsATS.201610-817OC 28222271

[45] Bennett RJ Fletcher S Conway N Barr C. The role of the general practitioner in managing age-related hearing loss: perspectives of general practitioners, patients and practice staff. BMC Fam Pract. (2020) 21(1):87. 10.1186/s12875-020-01157-2 32410580 PMC7226944

[46] Visvanathan R Amare AT Wesselingh S lnacio MC. General practitioner conduct of clinical services representing comprehensive geriatric assessment is associated with lower risk of mortality in older Australians receiving home care packages. Age Ageing. (2021) 50(4):1243–51. 10.1093/ageing/afaa272 33352580

[47] Guangyue J. Photometric Jump Theoretical Research on Family Doctor Information Platform Based on Hierarchical Diagnosis and Treatment System. Shanghai: Chinese People’s Liberation Army Naval Medical University (2016).

[48] Guohua Z Ping L Yunfei L. Study on hospital selection behavior of elderly patients with chronic diseases in China. Mod Chin Dr. (2023) 61(3):88–91. 10.3969/j.issn.1673-9701.2023.03.020

[49] Rücker G Schwarzer G Carpenter JR Schumacher M. Undue reliance on I(2) in assessing heterogeneity may mislead. BMC Med Res Methodol. (2008) 8:79. 10.1186/1471-2288-8-79 19036172 PMC2648991

